# The effect of baricitinib on pSTAT3 levels in IL-6- or IL-15-stimulated PBMCs isolated from patients with SLE

**DOI:** 10.3389/fimmu.2025.1675350

**Published:** 2025-10-21

**Authors:** Gábor J. Szebeni, Nikolett Gémes, Patrícia Neuperger, Enikő Szabó, József Á. Balog, Dániel Honfi, Attila Balog, Gergely Toldi

**Affiliations:** ^1^ Laboratory of Functional Genomics, Core Facility, HUN-REN Biological Research Centre, Szeged, Hungary; ^2^ Department of Internal Medicine, Hematology Centre, Faculty of Medicine, University of Szeged, Szeged, Hungary; ^3^ Department of Rheumatology and Immunology, Faculty of Medicine, Albert Szent-Gyorgyi Health Centre, University of Szeged, Szeged, Hungary; ^4^ Liggins Institute, University of Auckland, Auckland, New Zealand

**Keywords:** baricitinib, pSTAT3, systemic lupus erythematosus, multicolor flow cytometry, JAK/STAT pathway

## Abstract

**Introduction:**

Systemic lupus erythematosus (SLE) is a systemic autoimmune disease marked by multi-organ inflammation. Its pathogenesis involves profound T-cell dysfunction, autoreactive B-cell activation, impaired CD8^+^ T-cell responses, myeloid cell abnormalities, and dysregulated cytokine secretion. Central to cytokine-driven immune activation is the Janus kinase/signal transducer and activator of transcription (JAK/STAT) signaling pathway. Baricitinib, a selective oral JAK1/2 inhibitor approved for rheumatoid arthritis, has been extensively studied in SLE.

**Methods:**

We aimed to investigate STAT3 phosphorylation in CD4^+^ and CD8^+^ T cells and CD11b^+^ myeloid cells from patients with SLE using single-cell flow cytometry of peripheral blood mononuclear cells (PBMCs) stimulated *ex vivo* with interleukin-6 (IL-6) or IL-15. We quantified pSTAT3 induction and assessed the inhibitory effect of baricitinib.

**Results:**

Despite long-term immunomodulators, significant STAT3 activation was observed in T cells and myeloid cells upon IL-6 or IL-15 stimulation in patients with SLE. Baricitinib effectively inhibited STAT3 phosphorylation in these cell types, though its inhibitory effect was notably weaker following IL-15 stimulation compared to IL-6. Notably, baricitinib did not affect the proportion of interferon-γ (IFN-γ)- or IL-17-expressing cells.

**Conclusion:**

These findings highlight the cell-type and cytokine-specific effects of baricitinib and demonstrate its capacity to dampen IL-6- and IL-15-mediated STAT3 activation in key immune cell subsets. Our results support a precision medicine approach to JAK inhibition in SLE and reinforce the potential of baricitinib in modulating key inflammatory pathways.

## 1 Introduction

Systemic lupus erythematosus (SLE) is an archetypal systemic autoimmune disorder characterized by multi-organ inflammation and significant morbidity. Over recent decades, profound T cell-driven disturbances—including both numerical and functional abnormalities—have been established as central to its pathogenesis. Notably, CD4^+^ T helper subsets serve as key drivers of autoreactive B-cell activation, autoantibody production, and tissue injury across organs such as skin and kidneys. Conversely, CD8^+^ T cells, despite their cytolytic capacity, often display functional impairment in patients with SLE, compromising immune regulation. In parallel, dysregulated myeloid cell activation contributes to inflammation and organ damage via aberrant cytokine secretion and infiltrative damage (1–4).

At the molecular level, the Janus kinase/signal transducer and activator of transcription (JAK/STAT) pathway is pivotal to cytokine-mediated immune activation. Baricitinib, an oral selective JAK1/2 inhibitor, has regulatory approval for indications including rheumatoid arthritis (RA), alopecia areata, atopic dermatitis, and severe COVID-19 (4, 5). Its inhibitory potency is reflected in low nanomolar IC_50_ values for JAK1 and JAK2 (approximately 5–6 nM), with significantly lower activity against JAK3 and TYK2 (6).

In SLE, multiple pathologic cytokines—such as type I interferons (IFNs), interleukin-6 (IL-6), IL-15, IL-2, IL-17, and IL-23—utilize JAK/STAT signaling. Among these, IL-6 and IL-15 both stimulate STAT3 phosphorylation in a JAK1-dependent manner, whereas JAK2-mediated STAT3 activation is essential for myeloid differentiation (7). Patients with active or refractory SLE commonly exhibit elevated IL-6 levels, accompanied by increased numbers and persistent tissue infiltration of activated CD4^+^ and CD8^+^ T cells, as well as myeloid cells. STAT3 phosphorylation (pSTAT3) in these cells has emerged as a promising prognostic and predictive biomarker, correlating with disease activity in peripheral blood and affected tissues (1).

In preclinical lupus models, including MRL/lpr and NZB/W F1 mice, JAK inhibition has yielded beneficial effects: JAK2–STAT1 blockade reduced glomerular immune complex deposits, proteinuria, and inflammatory infiltrates; JAK1/STAT3 inhibition decreased disease severity and autoantibody levels (7).

In the clinical setting, recent trials have produced mixed results, highlighting both the rationale and caution surrounding baricitinib use in SLE. While its potency in modulating pSTAT3-driven inflammation underpins therapeutic rationale, concerns regarding infection risk and variable efficacy underscore the need for careful patient selection, biomarker-driven stratification, and monitoring (8, 9). A phase 2 randomized, placebo-controlled trial (24 weeks, 4 mg) demonstrated statistically significant reductions in overall disease activity, arthritis, and cutaneous manifestations compared with placebo (10). The subsequent SLE BRAVE I phase 3 trial confirmed SLE Responder Index 4 (SRI 4) response superiority (4 mg versus placebo) (11). However, the SLE BRAVE II mirrored trial failed to replicate these results (12). Meta-analysis of phase 2 and 3 datasets reinforced baricitinib’s modest efficacy (RR = 1.11 for SRI 4), though with modest increases in serious infections (8).

Taken together, these findings emphasize the critical role of JAK1/2-mediated cytokine signaling—especially IL-6 and IL-15–STAT3—in elevating activation phenotypes across T cells and myeloid cells in SLE. Nevertheless, mixed clinical results with baricitinib have underscored the importance of patient and pathway stratification.

Our aim was to investigate STAT3 phosphorylation in CD4^+^ T cells, CD8^+^ T cells, and CD11b^+^ myeloid cells using single-cell flow cytometry of PBMCs isolated from healthy controls and patients with SLE upon inducing a strong inflammatory response comparable to pathological conditions by IL-6 or IL-15 stimulation *ex vivo*. We then evaluated the anti-inflammatory, inhibitory effect of baricitinib across these cell types. These data are intended to support a stratified precision medicine approach in JAK based therapy for SLE.

## 2 Methods

### 2.1 Patient selection

Clinical details, including clinical characteristics and medications at the time of sampling, are summarized in **Table 1**. The age of patients with SLE was 45 (34.5–51) years, their disease duration was 8.5 (3–12) years, while their systemic lupus erythematosus disease activity index (SLEDAI) was 8 (6–10), corresponding to moderate disease activity (median, quartiles). The age of healthy controls (HC) was matched. Controls did not suffer from an inflammatory condition or infection at the time of sampling and at least 3 months prior, and they had a negative history for autoimmune conditions. Of note, ANA positivity was not tested in HC due to lack of clinical indication. All participants were women. Patients with SLE were diagnosed and classified according to the 2019 EULAR/ACR criteria (13). Written informed consent was obtained from all subjects, and our study was reviewed and approved by an independent ethical committee of the University of Szeged (ETT-TUKEB 149/PI/19). Laboratory studies and interpretations were performed on coded samples lacking personal and diagnostic identifiers. The study adhered to the tenets of the most recent revision of the Declaration of Helsinki.

**Table 1:** Main clinical characteristics and medications in patients with systemic lupus erythematosus and healthy individuals at the time of blood sampling.

Characteristics	Healthy individuals *n* = 10	Patients with SLE *n* = 10
Age, years	47.5 [41–49]	45 [34.5–51]
Gender, male/female	0/10	0/10
SLE duration, years	–	8.5 [3–12]
SLEDAI	–	8 [6–10]
ANA positivity at the time of diagnosis	–	10/10
ANA positivity at the time of sampling	–	9/10
aDNA positivity	–	9/10
Low complement 3 and 4	–	8/10
Lupus anticoagulant positivity	–	5/10
Photosensitivity	–	8/10
Raynaud phenomenon	–	9/10
Further skin symptoms*	–	6/10
Arthritis	–	9/10
Lymphadenomegaly	–	4/10
Splenomegaly	–	1/10
Hemolytic anemia	–	1/10
Leucopenia	–	2/10
Lymphopenia	–	3/10
Hydroxychloroquine	–	8/10
Methyl-prednisolone, 4 mg/day	–	10/10
Azathioprine	–	3/10
Methotrexate	–	1/10
Mycophenolate mofetil	–	3/10
CRP, mg/L	2.40 [BLD–4.15]	11.50 ^a^ [5.70–21.40]
ESR, mm/h	8 [6–14]	39 ^a^ [26–73]

Data are expressed as median [interquartile range]. ^a^ *p* < 0.05 vs. healthy individuals. Medications refer to therapy received at the time of sampling. *Further skin symptoms included discoid lupus volt, malar rash, and alopecia. SLE, systemic lupus erythematosus; SLEDAI, systemic lupus erythematosus disease activity index; ANA, anti-nuclear antibody; aDNA, anti-double-stranded DNA antibody; CRP, C-reactive protein; BLD, below the limit of detection; ESR, erythrocyte sedimentation rate, taken after 1 h.

### 2.2 PBMC isolation

Peripheral blood was drawn into Lithium-Heparin vacutainers (Beckton Dickinson, 4 × 10 mL tubes per participant for 40 mL of peripheral blood) at the outpatient clinic of the Department of Rheumatology and Immunology, University of Szeged, Szeged, Hungary. The isolation of peripheral blood mononuclear cells (PBMCs) was performed at the Biological Research Centre, Szeged, Hungary, within 1 h after sampling. PBMCs were isolated by Ficoll density gradient centrifugation as described previously by our group (14, 15).

### 2.3 Treatment of PBMCs

Cells (5 × 10^5^) were pelleted into 96-well plates in 90 μL of RPMI (Capricorn Scientific, Ebsdorfergrund, Germany) containing 0.3 g/L glutamine, 10% fetal calf serum (FCS) (Euroclone), 100 U/mL penicillin sodium salt, and 100 μg/mL streptomycin sulfate (Gibco). Baricitinib was dissolved in dimethyl sulfoxide (DMSO) 10 mM as a stock solution. Cells were treated with the indicated concentrations of baricitinib in 5 μL. The untreated control samples were kept in cell culture medium containing the DMSO vehicle as the same percentage of baricitinib. Samples were incubated for 15 min in 5% CO_2_ 37°C humidified thermostat (Sanyo). The stimulations were carried out with IL-6–100 ng/mL or IL-15–100 ng/mL (Sino Biological) based on literature data in 5 μL or with 5 μL cell culture medium for control samples (16–18). Samples were incubated for 15 min in a 5% CO_2_ 37°C humidified thermostat (Sanyo).

### 2.4 Fluorescent staining

Cells were suspended in 100 μL of PEB [0.5% bovine serum albumin (BSA) and 2 mM EDTA in phosphate-buffered saline (PBS)] (Miltenyi Biotec) and were centrifuged at 360*g* for 6 min, and the supernatant was discarded. Cells were labeled with the viability dye: Viobility 405/450 (Miltenyi Biotec) in 1:50 dilution in PBS containing 4% Fc receptor Blocker (True Stain, BioLegend) in 100 μL final volume at room temperature for 15 min. Cells were washed with 1 mL of IFB (2% FCS in PBS) and were centrifuged at 360*g* for 6 min, and the supernatant was discarded. Cells were labeled with the indicated antibodies in the **Supplementary Table** in 100 μL of IFB at room temperature in the dark for 20 min. One sample per subject was left unstained to set the autofluorescence. Cells were washed with 1 mL of IFB (2% FCS in PBS) and were centrifuged at 360*g* for 6 min, and the supernatant was discarded. Cells were resuspended in 100 μL of PEB. Samples were fixed with 100 μL of formaldehyde (3.7% stock, Miltenyi Biotec). Cells were gently suspended and incubated at room temperature for 20 min. Cells were washed with 1 mL of IFB (2% FCS in PBS) and were centrifuged at 360*g* for 6 min, and the supernatant was discarded. Cells were permeabilized by adding 500 μL of −80°C cold MetOH (fast) and placed into a −20°C freezer for 1 h. Cells were washed with 600 μL of PBS and were centrifuged at 360*g* for 6 min, and the supernatant was discarded. Cells were then washed with 1 mL of PBS containing 0.2% BSA and 0.1% Triton X-100 and were centrifuged at 360*g* for 6 min, and the supernatant was discarded. Cells were washed again with 1 mL of PBS and were centrifuged at 360*g* for 6 min, and the supernatant was discarded. Antibodies for intracellular staining were added in 100 μL of PBS containing 0.2% BSA and 0.1% Triton X-100 at room temperature, in the dark for 20 min. Cells were washed with 1 mL of IFB and were centrifuged at 360*g* for 6 min, and the supernatant was discarded. Cells were resuspended and acquired on a Cytoflex S flow cytometer equipped with 405-nm (channels: 450/45; 525/40; 610/20; 660/10), 488-nm (channels: 525/40; 690/50), 561-nm (channels: 610/20; 585/42; 690/50; 780/60), and 638-nm lasers (channels: 660/10; 712/25; 780/60) (Beckman Coulter).

### 2.5 Analysis

FCS files were analyzed using CytExpert 2.4 Software (Beckman Coulter). Manual gating was performed to gate on lymphocytes, single cells, living cells, CD3^+^ T cells, and CD3^−^ non-T cells. Subpopulations were gated as CD3^+^/CD8^+^ or CD3^+^/CD4^+^ T cells, and CD11b^+^ myeloid cells within the CD3^−^ population. Reporting channels were the following: CD4^+^pSTAT3^+^, CD8^+^pSTAT3^+^, CD11b^+^pSTAT3^+^, CD4^+^IL-17^+^, CD8^+^IL-17^+^, and CD11b^+^IL-17^+^.

### 2.6 Statistics

The proportions of cells expressing pSTAT3, IL-17, or IFN-γ were compared between treatment concentrations of baricitinib following stimulation with IL-6 or IL-15 in patients with SLE and in HC. Normally distributed datasets were compared with parametric, repeated-measures (RM) one-way analysis of variance (ANOVA). For non-parametric analysis, Friedman test was applied. All types of significance tests were corrected for multiple comparison by controlling the false discovery rate (FDR) with a two-stage Benjamini, Krieger, and Yekutieli approach with an FDR cutoff of 10%. Differences are considered significant at *p < 0.05; **p < 0.01; ***p < 0.001, and ****p < 0.0001. Green columns show arithmetic mean, and red bars specify standard error of the mean (SEM). Statistics were calculated using GraphPad Prism 9.3.1 software.

## 3 Results

### 3.1 Single-cell determination of responding CD4^+^ T cells, CD8^+^ T cells, and CD11b^+^ myeloid cells for IL-6 or IL-15 stimulation and baricitinib inhibition on STAT3 phosphorylation

**Figure 1:**
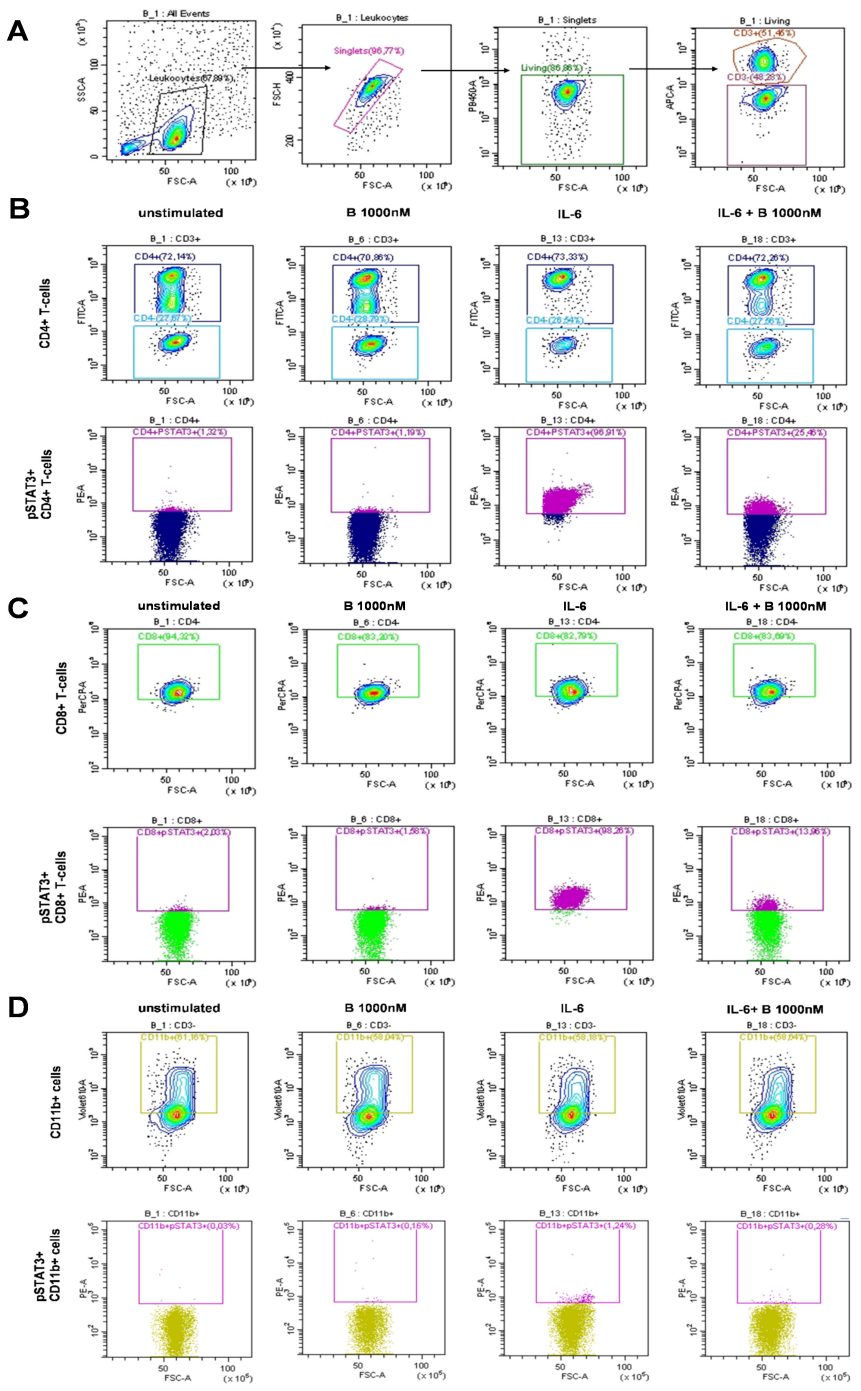
Representative examples of manual gating after IL-6 stimulation. **(A)** Leukocytes, single cells (singlets), live cells, and CD3^+^ or CD3^−^ cells were manually gated. Cells were left untreated or treated as follows: baricitinib 1,000 nM (B 1,000 nM), IL-6 (100 ng/mL), or treated with IL-6 + B 1,000 nM. **(B)** CD4^+^ T cells were gated within CD3^+^ T cells (top row). pSTAT3^+^ cells were gated within CD4^+^ T cells (bottom row). **(C)** CD8^+^ T cells were gated within CD4^−^ T cells (top row). pSTAT3^+^ cells were gated within CD8^+^ T cells (bottom row). **(D)** CD11b^+^ cells were gated within CD3^−^ cells (top row). pSTAT3^+^ cells were gated within CD11b^+^ cells (bottom row).

**Figure 2:**
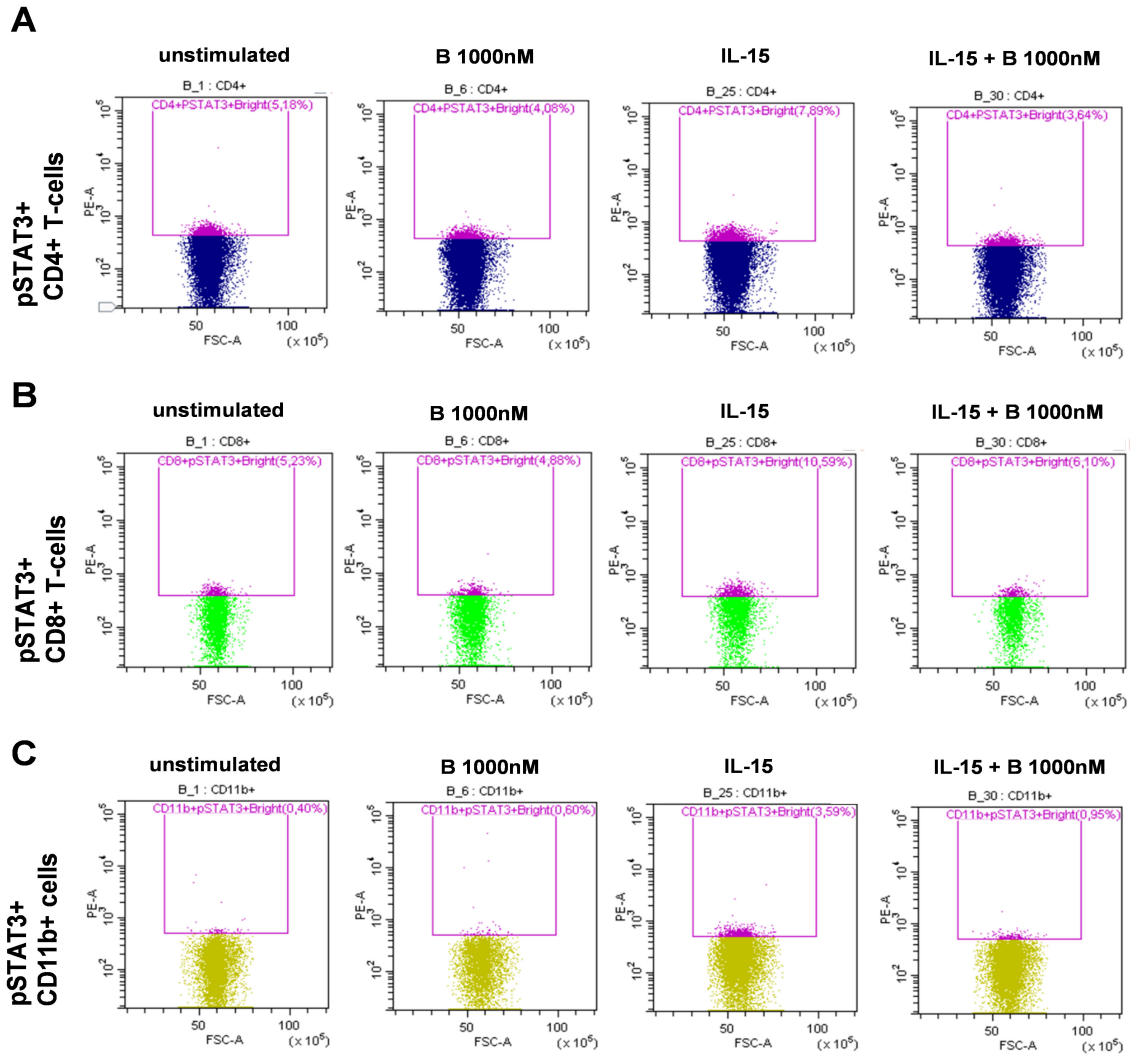
Representative examples of manual gating after IL-15 stimulation. Leukocytes, single cells (singlets), live cells, and CD3^+^ or CD3^−^ cells were manually gated as described in **Figure 1**. Cells were left untreated or treated as follows: baricitinib 1,000 nM (B 1,000 nM), IL-15 (100 ng/mL), or treated with IL-15 + B 1,000 nM. CD4^−^ T cells were gated within CD3^+^ T cells. CD8^+^ T cells were gated within CD4^−^ T cells. CD11b^+^ cells were gated within CD3^−^ cells. **(A)** pSTAT3^+^ cells were gated within CD4^+^ T cells. **(B)** pSTAT3^+^ cells were gated within CD8^+^ T cells. **(C)** pSTAT3^+^ cells were gated within CD11b^+^ cells.

PBMCs derived from patients with SLE were treated and assayed *ex vivo* freshly following isolation. Single-cell multicolor flow cytometry (MFC) was used to gate on leukocytes, single cells, living cells, and CD3^+^ T cells or CD3^−^ cells (**Figure 1A**). Within the CD3^+^ T-cell compartment, CD4^+^ T cells were gated (**Figure 1B**), or within the CD3^+^/CD4^−^ compartment, CD8^+^ T cells were gated (**Figure 1C**). The CD11b^+^ myeloid cells were gated within the CD3^−^ population (**Figure 1D**). MFC was used to measure the induction of STAT3 phosphorylation upon IL-6 stimulation and determine the degree of inhibition following baricitinib treatment. Representative gates are shown for the IL-6-mediated induction of pSTAT3 and to demonstrate the effect of 1,000 nM baricitinib in CD4^+^ T cells (**Figure 1B**), CD8^+^ T cells (**Figure 1C**), and CD11b myeloid cells (**Figure 1D**). MFC was used to measure the induction of STAT3 phosphorylation upon IL-15 stimulation and determine the degree of inhibition following baricitinib treatment. Representative gates are shown for the IL-15-mediated induction of pSTAT3 and to demonstrate the effect of 1,000 nM baricitinib in CD4^+^ T cells (**Figure 2A**), CD8^+^ T cells (**Figure 2B**), and CD11b myeloid cells (**Figure 2C**).

### 3.2 The effect of IL-6 and baricitinib on pSTAT3 expression

**Figure 3:**
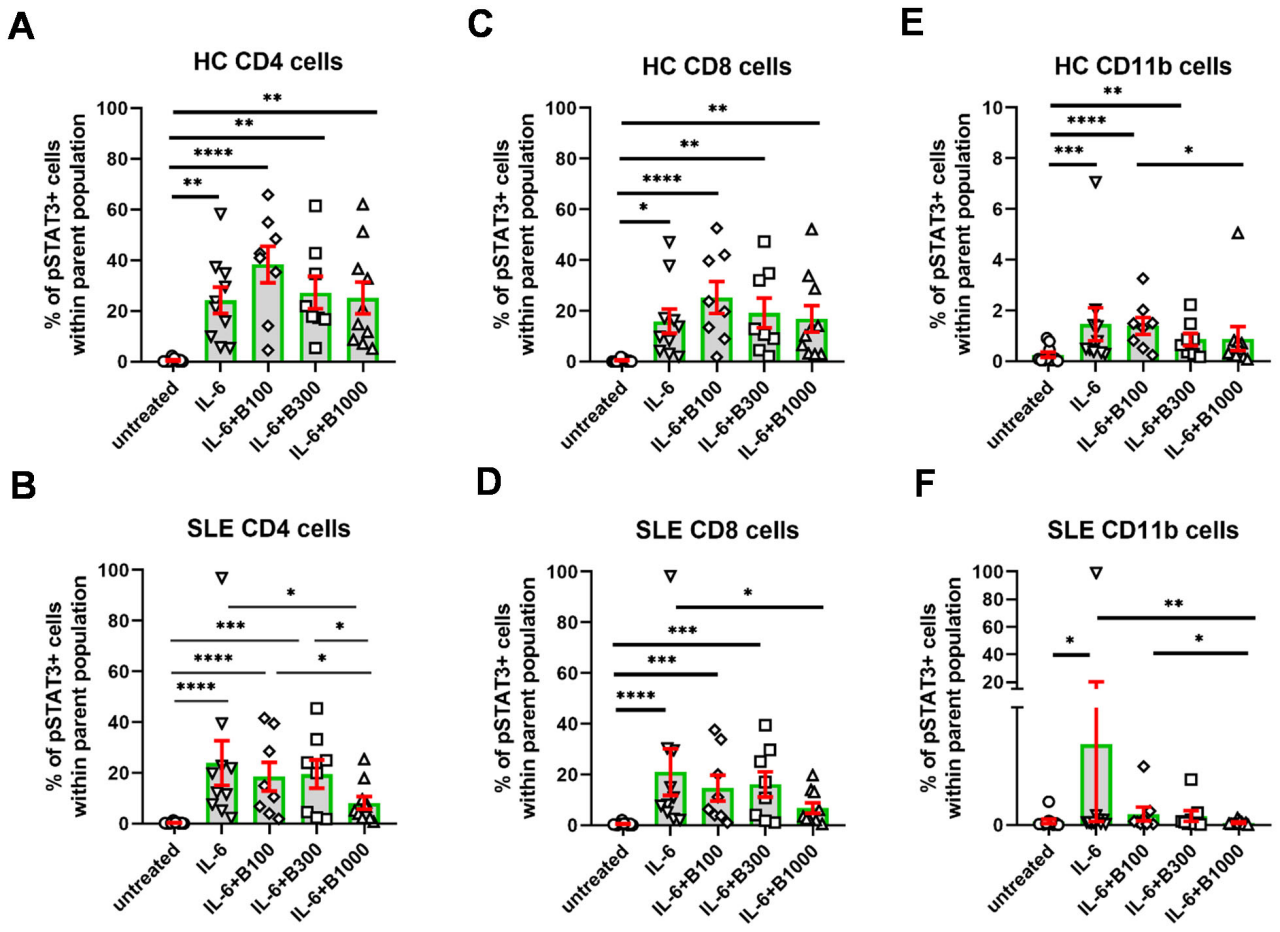
The percentage of pSTAT3^+^ cells following stimulation with IL-6. Baricitinib was tested to inhibit pSTAT3 induction at 100 (*n* = 8)/300 (*n* = 8)/1,000 (*n* = 10) nM in SLE-derived cells. Age- and gender-matched healthy controls (HC) were enrolled. **(A, B)** CD4^+^ T cells, **(C, D)**, CD8^+^ T cells, **(E, F)** CD11b cells upon IL-6 stimulation and baricitinib treatment. Data are shown as individual values gated within the parental CD4^+^, CD8^+^, or CD11b^+^ population, respectively. Green columns represent arythmetic mean and the red bars show SEM (standard error of the mean). *p<0.05, **p<0.01, ***p<0.001, ****p<0.0001.

The induction of pSTAT3 by IL-6 was similar in SLE (23.9% ± 8.8%) and in HC (24.1% ± 5.2%) both in CD4^+^ T cells (**Figures 3A, B**) and in CD8^+^ T cells (**Figures 3C, D**, SLE: 20.9% ± 9.1%; HC: 15.9% ± 4.7%). In contrast, SLE-derived CD11b^+^ myeloid cells responded to IL-6 induction with a higher extent (10.3% ± 9.8%) than HC-derived CD11b^+^ cells (1.5% ± 0.6%) (**Figures 3E, F**). However, the proportion of pSTAT3-expressing cells following stimulation with IL-6 was lowest in CD11b^+^ myeloid cells compared to CD4^+^ and CD8^+^ T lymphocytes (**Figure 3**). Baricitinib (B) did not reduce the proportion of pSTAT3-expressing cells in either subset in HC (**Figures 3A, C, E**). In contrast, the addition of B 1,000 nM resulted in a significant reduction of the proportion of pSTAT3-expressing cells in CD4^+^T cells (8.2% ± 2.5%), CD8^+^ T cells (6.8% ± 2.0%), and CD11b^+^ cells (0.3% ± 0.1%) in SLE (**Figures 3B, D, F**). Lower concentrations of baricitinib (B 100 nM and B 300 nM, *n* = 8 each) did not appear to alter the proportion of pSTAT3-expressing cells in SLE.

### 3.3 The effect of IL-15 and baricitinib on pSTAT3 expression

**Figure 4:**
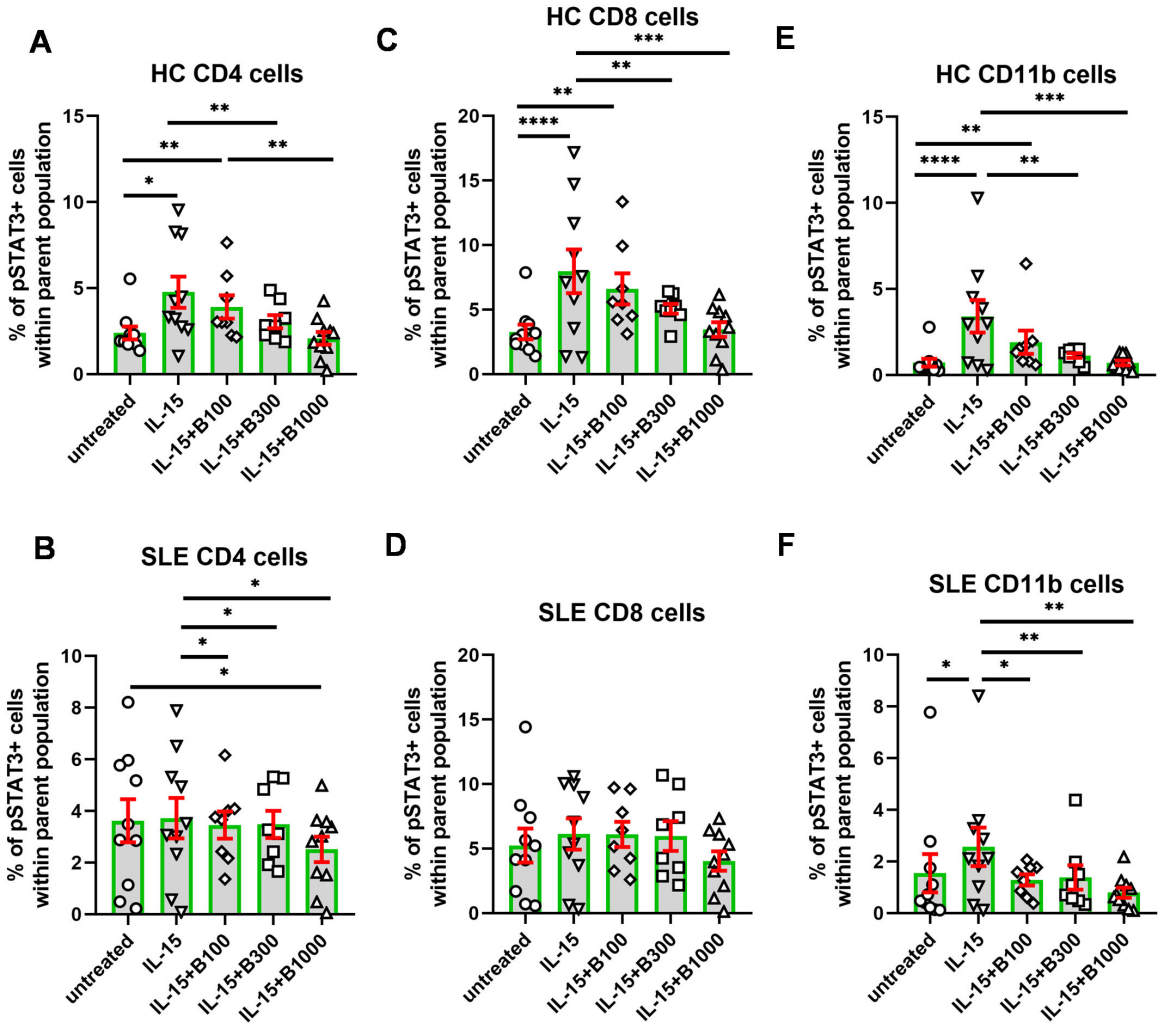
The percentage of pSTAT3^+^ cells following stimulation with IL-15. Baricitinib was tested to inhibit pSTAT3 induction in 100 (*n* = 8)/300 (*n* = 8)/1,000 (*n* = 10) nM in SLE-derived cells. Age- and gender-matched healthy controls (HC) were enrolled. **(A, B)** CD4^+^ T cells, **(C, D)**, CD8^+^ T cells, and **(E, F)** CD11b cells upon IL-15 stimulation and baricitinib treatment. Data are shown as individual values gated within the parental CD4^+^, CD8^+^, or CD11b^+^ population, respectively. Green columns represent arythmetic mean and the red bars show SEM (standard error of the mean). *p<0.05, **p<0.01, ***p<0.001, ****p<0.0001.

In contrast with IL-6, the degree of activation in terms of the increase of pSTAT3 was lower following IL-15 stimulation in each subset under investigation (**Figure 4**). The gating for pSTAT3^+^ cells was modified because of the lower extent of induction. Interestingly, induction of pSTAT3 upon IL-15 was higher in HC than in SLE-derived cells. Namely, the mean of the percentage of reactive cells (subtracting the mean of unstimulated background) upon IL-15 stimulation was 2.4% in CD4^+^, 4.7% in CD8^+^, and 2.7% in CD11b^+^ cells in HC and 0.1% in CD4^+^, 0.9% in CD8^+^, and 1.0% in CD11b^+^ cells in SLE, respectively (**Figure 4**). Baricitinib (100 nM) was effective in SLE CD4^+^ and SLE CD11b^+^ cells, while 300 nM baricitinib decreased the proportion of pSTAT3-expressing cells in all conditions significantly except for CD8^+^ SLE-derived T cells.

No differences were identified in the proportion of IFN-γ- or IL-17-expressing cells following baricitinib treatment in SLE or HC in any cell subset following IL-6 or IL-15 stimulation (**Supplementary Figure 1**). There were also no differences in pSTAT3 expression identified in the Th17.1 cell subset in SLE or HC.

### 3.4 Individual response rate to baricitinib

**Figure 5:**
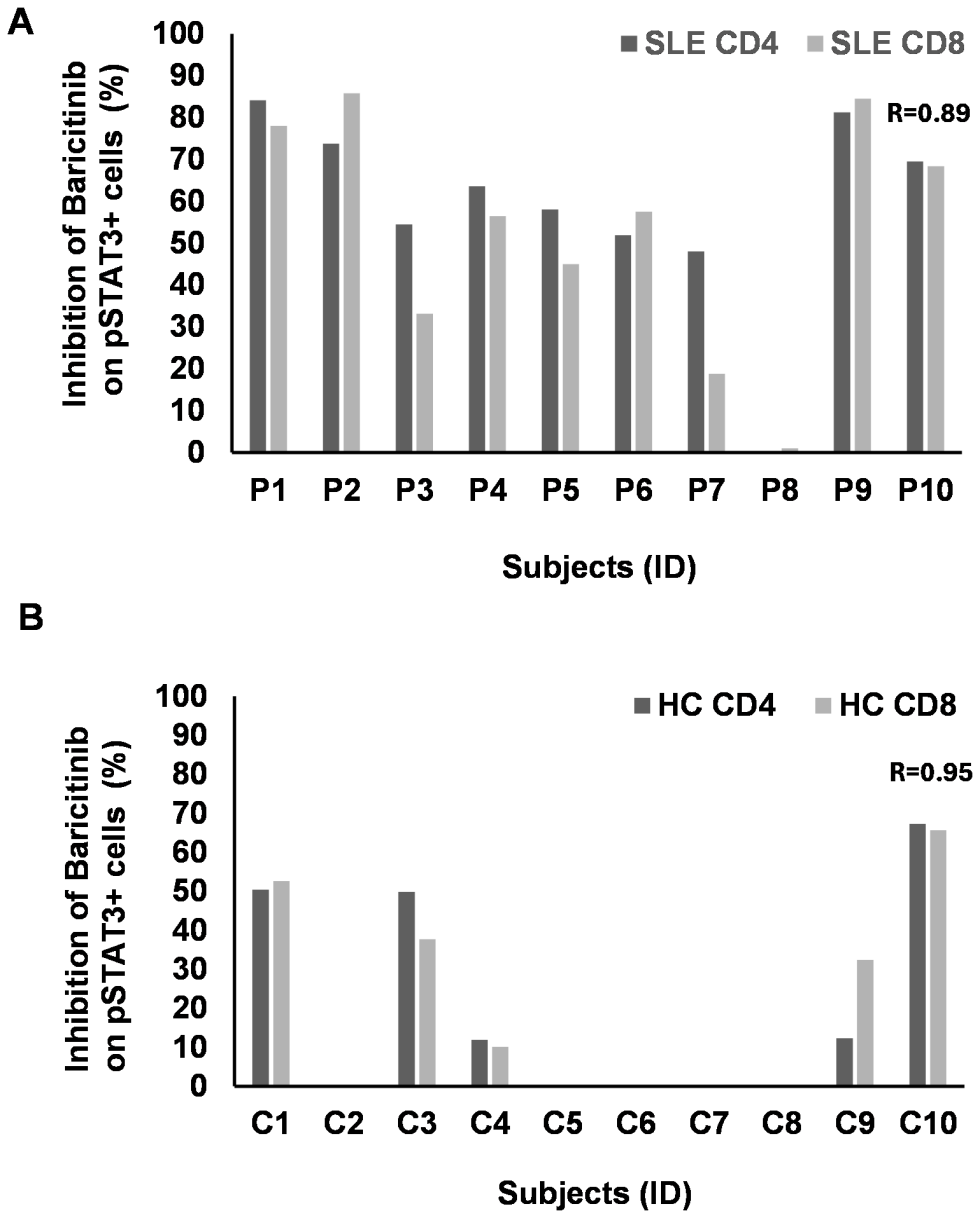
The rate of inhibition of baricitinib on pSTAT3 induction by IL-6 individually. PBMCs of patients with SLE (*n* = 10) and healthy controls (HC, *n* = 10) were stimulated by IL-6. Baricitinib (1,000 nM) was added to block JAK-mediated STAT3 phosphorylation. The percentage of inhibition was calculated from the flow cytometry data for each subject separately. No graph represents no inhibition. **(A)** CD4 (dark gray) or CD8 T cells (light gray) from patients with SLE were analyzed upon IL-6 stimulation and baricitinib treatment. The rate of inhibition, where present, strongly correlates between CD4 and CD8 T cells (Pearson’s *R* = 0.89). **(B)** CD4 (dark gray) or CD8 T cells (light gray) from HC were analyzed upon IL-6 stimulation and baricitinib treatment. The rate of inhibition, where present, strongly correlates between CD4 and CD8 T cells (Pearson’s *R* = 0.95). Equation: 100 − (stimulated + B 1,000 nM/stimulated * 100).

We analyzed above the single-cell flow cytometric data as a pool of subjects within a cohort to calculate statistics for the entire experimental group. In order to obtain data about the individual effect of baricitinib, a personalized medicine-based approach was applied. The individual rate of inhibition of baricitinib was counted as between the stimulated state versus baricitinib-treated cells for each subject individually. There was a clear concordance between the decline of pSTAT3^+^/CD4^+^ and pSTAT3^+^/CD8^+^ T cells (*R* = 0.89) after IL-6 induction (**Figure 5A**). Of 10 patients, 8 showed higher inhibition of pSTAT3 upon IL-6 stimulation than 50% in CD4^+^ T cells in SLE. Six patients reached 50% inhibition of pSTAT3 upon IL-6 stimulation in CD8^+^ T cells in SLE (**Figure 5A**). HC showed moderate inhibition of pSTAT3 upon IL-6 stimulation in both CD4 and CD8^+^ T cells, and only three of eight cases were inhibited up to 40%–50% (**Figure 5B**). There was no concordance between SLE and HC-derived CD11b^+^ cells in terms of pSTAT3 inhibition following the addition of IL-6. One patient with SLE (P8) was refractory to baricitinib, albeit IL-6 induction was approximately 2% in CD4^+^ and CD8^+^ T cells and 0.2% in CD11b^+^ cells, suggesting the activation in other inflammatory pathways involved in the pathomechanisms of SLE in that patient.

**Figure 6:**
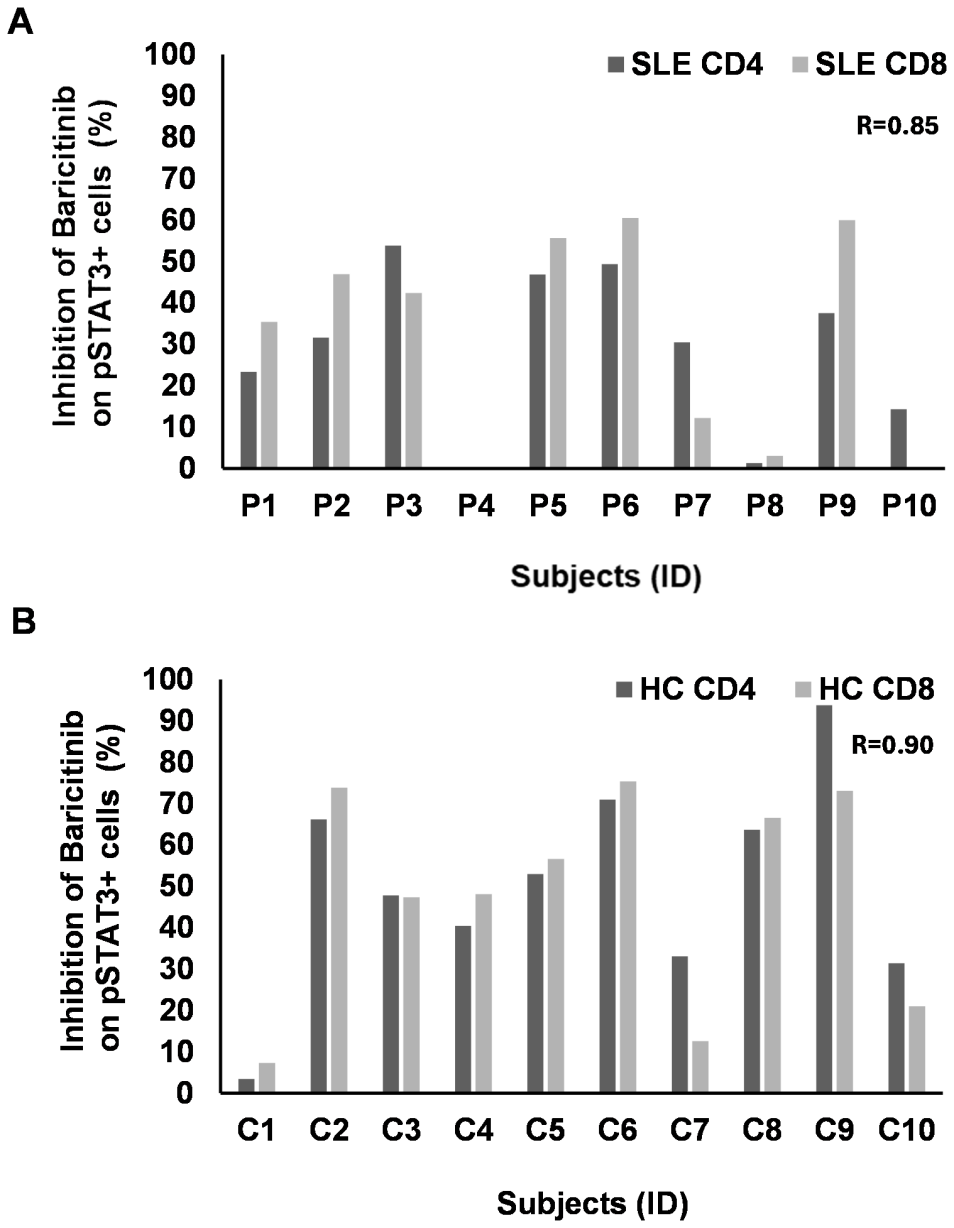
The rate of inhibition of baricitinib on pSTAT3 induction by IL-15 individually. PBMCs of patients with SLE (*n* = 10) and healthy controls (HC, *n* = 10) were stimulated by IL-15. Baricitinib (1,000 nM) was added to block JAK-mediated STAT3 phosphorylation. The percentage of inhibition was calculated from the flow cytometry data for each subject separately. No graph represents no inhibition. **(A)** CD4 (dark gray) or CD8 T cells (light gray) from patients with SLE were analyzed upon IL-6 stimulation and baricitinib treatment. The rate of inhibition, where present, strongly correlates between CD4 and CD8 T cells (Pearson’s *R* = 0.85). **(B)** CD4 (dark gray) or CD8 T cells (light gray) from HC were analyzed upon IL-6 stimulation and baricitinib treatment. The rate of inhibition, where present, strongly correlates between CD4 and CD8 T cells (Pearson’s *R* = 0.90). Equation: 100 − (stimulated + B 1,000 nM/stimulated * 100).

There was a clear concordance between the decline of pSTAT3^+^/CD4^+^ and pSTAT3^+^/CD8^+^ T cells (*R* = 0.85) after IL-15 induction in SLE (**Figure 6A**). However, the rate of inhibition of pSTAT3 was much lower following IL-15 induction in CD4^+^ and CD8^+^ SLE-derived samples compared to the inhibition after IL-6 stimulation. Out of 10 patients, 7 showed inhibition of pSTAT3 upon IL-15 stimulation between 20% and 50% in CD4^+^ T cells in SLE. Out of 10 patients, 6 showed inhibition of pSTAT3 upon IL-15 stimulation between 20% and 50% in CD8^+^ T cells in SLE (**Figure 6A**). CD4^+^ and CD8^+^ T cells of two cases (P4 and P8) were resistant to baricitinib in SLE (**Figure 6A**). HC showed clear correlation (*R* = 0.90) between CD4^+^ and CD8^+^ T cells with robust inhibition of pSTAT3 upon IL-15 stimulation of approximately 50% inhibition or above, in both CD4 and CD8^+^ T cells, in 8 out of 10 subjects (**Figure 6B**). Similar to IL-6, there was no concordance between SLE and HC-derived CD11b^+^ cells in terms of pSTAT3 inhibition following IL-15 induction. Although the rate of inhibition in these CD11b^+^ myeloid cells was among the highest, two SLE-derived samples reached 50% inhibition and six SLE-derived cells were inhibited between 60% and 80%.

## 4 Discussion

The potential use of baricitinib in SLE has recently been suggested by several studies. Promising results were obtained regarding its clinical efficacy and its impact on various inflammatory mediators. Using Olink technology, Dörner et al. demonstrated that baricitinib treatment in 239 patients with SLE significantly reduced levels of several pro-inflammatory plasma markers, including CCL9, CXCL10, CD137, PD-L1, IL-6, and IL-12β (2). Additionally, Allam et al. and Zaidi et al. each reviewed three clinical trials involving a total of 1,849 patients and found that baricitinib significantly outperformed placebo in achieving SRI-4 response. Furthermore, baricitinib was also superior in achieving SLEDAI-2K-defined remission of arthritis or rash (3, 8).

In a Phase 2 clinical trial, the 4-mg dose, but not the 2-mg dose of baricitinib led to significant improvements in the signs and symptoms of active SLE in patients whose disease was inadequately controlled by standard therapy (10). However, owing to safety concerns associated with chronic use of another JAK inhibitor, tofacitinib, the Food and Drug Administration (FDA) restricted the approved baricitinib dose to 2 mg/day. This corresponds to a steady-state plasma concentration of 9 ng/mL (19).

Morand et al. published the results of a Phase 3 multicenter, double-blind, placebo-controlled randomized trial, in which patients with active SLE received either 4 mg (*n* = 252) or 2 mg (*n* = 255) of baricitinib daily for 52 weeks. A significantly higher proportion of patients receiving 4 mg of baricitinib achieved the SRI-4 response (11). Yin et al., in a meta-analysis of these trials, concluded that both 4-mg and 2-mg doses were more effective in reducing disease activity among a subpopulation of patients receiving baseline glucocorticoid therapy (≥10 mg/day of prednisone or equivalent) (20). Shah et al. also reviewed four clinical trials and confirmed the superiority of baricitinib over placebo, while emphasizing the importance of careful patient selection and vigilant monitoring for adverse effects (9).

In addition to key cytokines implicated in SLE pathogenesis, such as type I IFNs, IL-2, and IL-17, other mediators contribute significantly to disease activity and severity. While treatments like anifrolumab (a type I IFN receptor blocker) and B-cell-targeting agents such as rituximab, obinutuzumab, and belimumab have shown efficacy in SLE, a considerable proportion of patients remain partial or non-responders (21, 22). Continued research into novel therapies, including those targeting less-studied cytokines such as IL-6 and IL-15 and the JAK/STAT pathway, is therefore essential for improving patient outcomes and may contribute to the development of personalized therapeutic strategies in SLE in the future. Our study focused on a homogeneous population of patients with SLE with moderate disease activity. Our findings support the presence of significant immune activation in peripheral blood immune cells, even among patients with moderate SLE activity. All participants were on long-term immunomodulators at the time of sampling. We demonstrated that baricitinib modulates STAT3 phosphorylation in CD4^+^ and CD8^+^ T cells, as well as CD11b^+^ myeloid cells, following stimulation with IL-6 or IL-15. Future studies will be necessary to evaluate specific concentrations of baricitinib in terms of both inhibitory efficacy and potential cytotoxic effects.

Most current drug development efforts target patients with severe active SLE. However, our study suggests that baricitinib may also offer therapeutic benefit in cases of moderate disease activity. Nonetheless, because of the small sample size (*n* = 10 per group) and variability in intracellular fluorescence intensity as measured by flow cytometry, these results should be interpreted with caution. This is particularly true for CD11b^+^ myeloid cells, which represented the smallest population within the analyzed subsets compared to CD4^+^ and CD8^+^ T cells. Interestingly, CD11b^+^ myeloid cells from patients with SLE showed stronger pSTAT3 activation upon IL-6 stimulation compared to HC, underscoring the relevance of non-lymphoid cell subsets to the contribution of the pathogenesis of chronic inflammation in SLE.

We examined whether the observed variability in the percentage of pSTAT3^+^ cells following IL-6 or IL-15 stimulation might be explained by potential correlations with clinical parameters, disease activity, or medical history. Of note, the variability does not seem to be associated with specific clinical or therapeutic characteristics of patients and likely reflects individual sensitivities to the cytokine stimulation applied. This also applies to the patient in our cohort who was ANA negative at the time of sample collection for our study but had been ANA positive at the time of diagnosis. Of note, this patient had received rituximab during their earlier course of the disease. The biological relevance of our findings also warrants cautious interpretation. The reduced responsiveness to IL-15 in SLE compared to HC is surprising, but in line with previous studies suggesting that immune cells demonstrate enhanced synthesis of IL-15 in SLE, with a poor response to this cytokine by different leucocyte subsets. This abnormal function of IL-15 may contribute to the pathogenesis of SLE (23, 24). Although IL-15 is not a potent inducer of pSTAT3 expression and results seen with IL-15 stimulation were not fully replicated with IL-6, our data still support the potential role of these cytokines in SLE pathogenesis and their viability as therapeutic targets. Future studies involving larger and more diverse SLE populations, including those with higher disease activity and varying organ involvement, are necessary to validate these findings and clarify the mechanisms of action of baricitinib.

In summary, our study demonstrates the cellular effects of baricitinib on STAT3 phosphorylation in key immune subsets, CD4^+^, CD8^+^ T cells, and CD11b^+^ myeloid cells, following IL-6 and IL-15 stimulation. These results support the therapeutic potential of baricitinib in modulating IL-6- and IL-15-driven inflammatory pathways in SLE.

## Data Availability

The raw data supporting the conclusions of this article will be made available by the authors, without undue reservation.

## Supplementary material

Supplementary Figure 1 No differences were identified in the proportion of IL-17-expressing CD4^+^, CD8^+^, or CD11b^+^ cells following baricitinib treatment in healthy controls (HC, *n* = 10) or patients with SLE (*n* = 10) upon IL-6 or IL-15 stimulation.
