# Supplementary figures and images for: The effect of baricitinib on pSTAT3 levels in IL-6- or IL-15-stimulated PBMCs isolated from patients with SLE
Source: Front Immunol. 2025 Oct 21;16:1675350. doi: 10.3389/fimmu.2025.1675350 (PMC12583204; doi:10.3389/fimmu.2025.1675350)

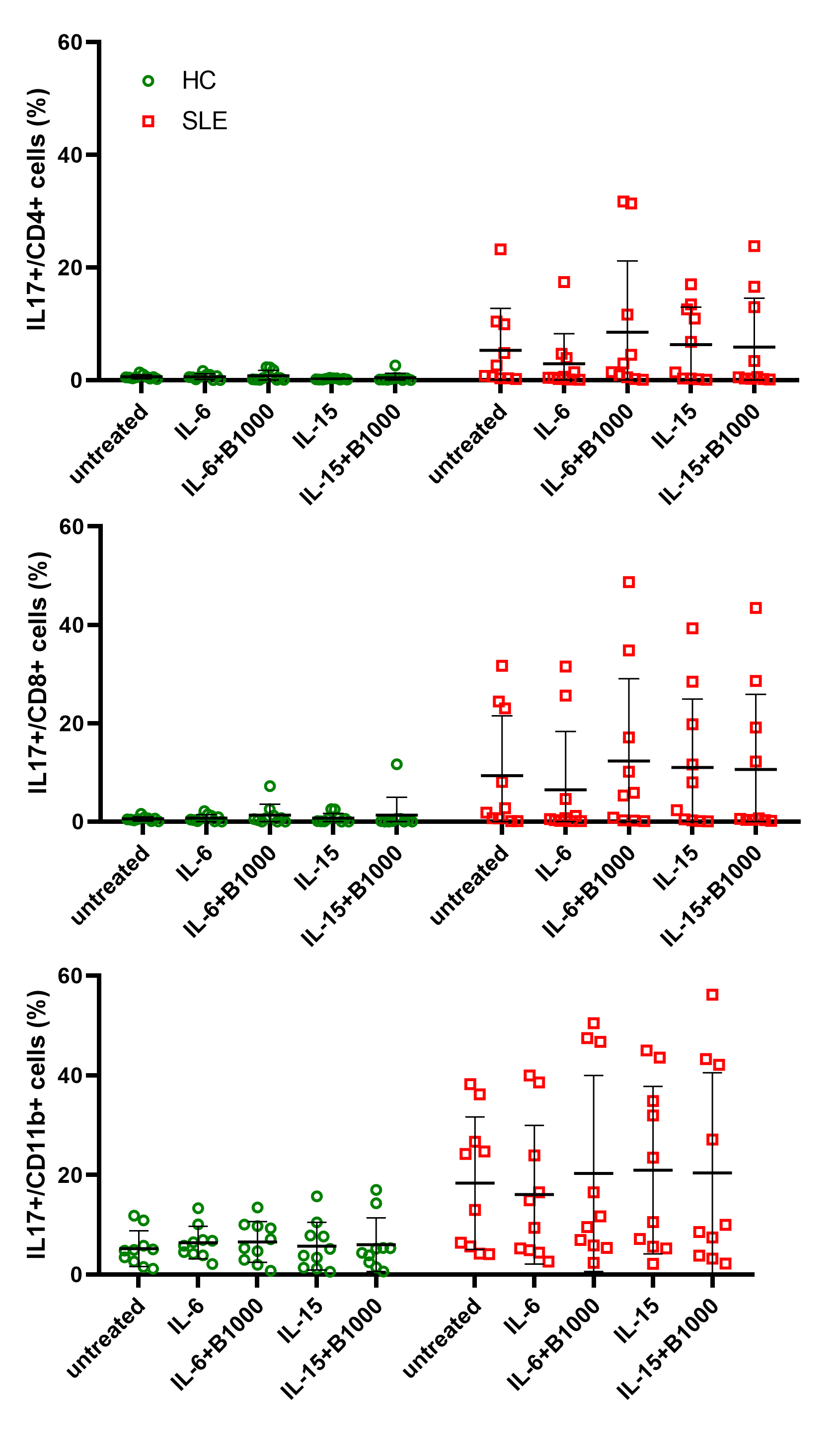

Supplement: Supplementary Figure 1 — No differences were identified in the proportion of IL-17-expressing CD4+, CD8+, or CD11b+ cells following baricitinib treatment in healthy controls (HC, n = 10) or patients with SLE (n = 10) upon IL-6 or IL-15 stimulation. [file Image1.tif]
