# Supplementary material for: The effect of baricitinib on pSTAT3 levels in IL-6- or IL-15-stimulated PBMCs isolated from patients with SLE
Source: Front Immunol. 2025 Oct 21;16:1675350. doi: 10.3389/fimmu.2025.1675350 (PMC12583204; doi:10.3389/fimmu.2025.1675350)
Supplement: Supplementary file 2 [file Table1.docx]

**Supplementray Table 1.** List of the antibodies used in the study.

| **Target** | **Clone** | **Fluorophore** | **Vendor** |
| --- | --- | --- | --- |
| anti-CD3 | OKT-3 | APC | Biolegend |
| anti-CD4 | REA623 | VioBright515 | Miltenyi |
| anti-CD8a | RPAT8 | PerCP | Biolegend |
| anti-CD11b | ICRF44 | Brilliant Violet 605 | Biolegend |
| anti-IL17-A | eBio64DEC17 | eFluor 506 | Thermo Fisher |
| anti-pSTAT3 | 13A3-1 | PE | Biolegend |
| anti-IFNγ | REA600 | APC Vio770 | Miltenyi |
